# Second Messenger Signaling in *Bacillus subtilis*: Accumulation of Cyclic di-AMP Inhibits Biofilm Formation

**DOI:** 10.3389/fmicb.2016.00804

**Published:** 2016-05-25

**Authors:** Jan Gundlach, Hermann Rath, Christina Herzberg, Ulrike Mäder, Jörg Stülke

**Affiliations:** ^1^Department of General Microbiology, Institute for Microbiology and Genetics, Georg-August-University GöttingenGöttingen, Germany; ^2^Interfaculty Institute for Genetics and Functional Genomics, University Medicine GreifswaldGreifswald, Germany

**Keywords:** biofilm formation, c-di-AMP, phosphodiesterase, SinR, *Bacillus subtilis*

## Abstract

The Gram-positive model organism *Bacillus subtilis* produces the essential second messenger signaling nucleotide cyclic di-AMP. In *B. subtilis* and other bacteria, c-di-AMP has been implicated in diverse functions such as control of metabolism, cell division and cell wall synthesis, and potassium transport. To enhance our understanding of the multiple functions of this second messenger, we have studied the consequences of c-di-AMP accumulation at a global level by a transcriptome analysis. C-di-AMP accumulation affected the expression of about 700 genes, among them the two major operons required for biofilm formation. The expression of both operons was severely reduced both in the laboratory and a non-domesticated strain upon accumulation of c-di-AMP. In excellent agreement, the corresponding strain was unable to form complex colonies. In *B. subtilis*, the transcription factor SinR controls the expression of biofilm genes by binding to their promoter regions resulting in transcription repression. Inactivation of the *sinR* gene restored biofilm formation even at high intracellular c-di-AMP concentrations suggesting that the second messenger acts upstream of SinR in the signal transduction pathway. As c-di-AMP accumulation did not affect the intracellular levels of SinR, we conclude that the nucleotide affects the activity of SinR.

## Introduction

Many bacteria are able to choose between a variety of different lifestyles. Depending on the growth state, the Gram-positive soil bacterium *Bacillus subtilis* has a large repertoire of potential choices. Vegetatively growing cells may be either motile to explore their environment for nutrients or sessile in biofilms. In the transition between logarithmic growth and the stationary phase, *B. subtilis* may secrete extracellular enzymes to degrade polymeric nutrients (the miner activity) or become competent for the uptake of foreign DNA. Finally, if no other option is left, stationary phase cells may sporulate or exhibit cannibalistic behavior (López and Kolter, 2010). To make sure that only a single chosen pathway is activated, the genes encoding the factors for the different lifestyles need to be tightly controlled.

In *B. subtilis*, biofilm formation and motility are two mutually exclusive lifestyles, and the choice between them is regulated at the levels of protein activities and gene expression (Vlamakis et al., 2013). In biofilm forming cells, the EpsE biofilm protein acts like a molecular clutch that arrests flagellar rotation by separating the cytoplasmic FliG motor from the MotA–MotB stator (Blair et al., 2008). Regulation at the level of gene expression is achieved by the master regulator SinR that binds to the promoter regions of two major operons required for biofilm formation, the *tapA-sipW-tasA* operon and the 15 gene *epsA-O* operon (Chu et al., 2006). The former operon encodes the amyloid-like fiber protein TasA and the proteins required for its export and assembly (Romero et al., 2014). The *eps* operon encodes the enzymes for the synthesis of the extracellular polysaccharide matrix for the biofilm, most likely poly-*N*-acetylglucosamine (Roux et al., 2015). The DNA-binding activity of SinR, in turn, is governed by protein–protein interactions between SinR and two antagonist proteins, SinI and SlrR (Vlamakis et al., 2013). SlrR, on the other hand, represses the expression of motility and autolysin genes when forming the complex with SinR. Thus, the formation of the SlrR–SinR complex results in the expression of biofilm genes and in the repression of motility genes (Cozy et al., 2012). At low SlrR levels, free SinR can repress the biofilm genes, and the motility genes are expressed under these conditions (Chai et al., 2009). Thus, the expression of the two sets of genes is mutually exclusive (Vlamakis et al., 2013). So far, it is unknown whether the interactions between SinR and its antagonists are regulated by additional factors. The regulation of motility and biofilm formation by these subtle protein–protein interactions differs even between individual cells of a seemingly homogeneous population of growing cells: each cell has to make an individual choice (Lopez et al., 2009; Diethmaier et al., 2011). Mutants that result in homogeneity, i.e., uniform modes of biofilm and motility gene expression in a culture, are excellent tools to study the signaling upstream of the SinR master regulator. Very recently, even the expression of SinR was shown to be heterogeneous (Ogura, 2016). We have recently shown that the phosphodiesterase YmdB is required for heterogeneity and for the expression of biofilm genes; in a *ymdB* mutant all cells express exclusively the motility genes (Diethmaier et al., 2011).

In most organisms that choose between motile and sessile lifestyles, so-called second messengers are involved in the decision-making. In *Escherichia coli* and many other Gram-negative bacteria, cyclic di-GMP stimulates biofilm formation and inhibits motility (Hengge, 2009; Römling et al., 2013). In *B. subtilis*, c-di-GMP is also present; however, the molecule is not involved in the control of biofilm formation (Gao et al., 2013). In contrast to enteric bacteria, Gram-positive bacteria also possess the second messenger cyclic di-AMP. This nucleotide has been implicated in several cellular processes such as cell division and cell wall synthesis, potassium homeostasis, and metabolism (for review see Corrigan and Gründling, 2013; Commichau et al., 2015). In *B. subtilis*, this second messenger can be synthesized by three distinct diadenylate cyclases (CdaA, CdaS, and DisA) and degraded by two phosphodiesterases (GdpP and PgpH). Interestingly, c-di-AMP is essential for the growth of *B. subtilis*, but at high concentrations it becomes toxic (Mehne et al., 2013; Gundlach et al., 2015b). The search for targets of c-di-AMP in *B. subtilis* has identified a subunit of a potassium transporter, KtrC, a PII-like signaling protein, DarA, and a riboswitch that is also engaged in the regulation of the expression of a potassium transporter (Corrigan et al., 2013; Nelson et al., 2013; Gundlach et al., 2015a). Importantly, none of the known targets explains the essentiality and toxicity of c-di-AMP.

To improve our understanding of c-di-AMP-mediated signal transduction in *B. subtilis*, we have compared the global gene expression patterns of a wild type strain and a strain that accumulates c-di-AMP due to a deletion of the two phosphodiesterases. Our results indicate that accumulation of c-di-AMP inhibits biofilm formation, and that this second messenger seems to affect the activity of the SinR transcription factor.

## Materials and Methods

### *B. subtilis* Strains and Growth Conditions

The *B. subtilis* strains used in this work are listed in **Table 1**. They are derived from the laboratory wild type strain 168 or from the non-domesticated wild type strain NCIB3610. *B. subtilis* was grown in LB medium or in Spizizen minimal medium containing glucose and glutamate as sources of carbon and nitrogen, respectively (Commichau et al., 2008). The medium was supplemented with auxotrophic requirements (at 50 mg/l). SP, CSE, YT, and MSgg (Branda et al., 2001) plates were prepared by the addition of 17 g Bacto agar/l (Difco) to the medium. To transfer mutations into the background of the non-domesticated wild-type strain NCIB3610, SPP1-mediated phage transduction was used as described previously (Diethmaier et al., 2011). Transductants were selected on CSE glucose and YT plates containing tetracyclin (Tc 12.5 μg/ml), spectinomycin (Spc 150 μg/ml), or erythromycin plus lincomycin (Em 2 μg/ml and Lin 25 μg/ml).

**Table 1:** Bacterial strains used in this study.

Strain	Genotype	Reference
168	*trpC2*	Laboratory collection
8G5	Δ*sinR*::*tet*	O. Kuipers
NCIB3610	wild type	Laboratory collection
GP736	*trpC2*Δ*sinR*::*tet*	8G5 → 168
GP921	NCIB3610 Δ*ymdB* ::*spc*	Diethmaier et al., 2011
GP998	*trpC2* Δ*gdpP*::*spc*	Mehne et al., 2013
GP1562	NCIB3610 Δ*sinR::spc*	TMB079 → NCIB3610
GP1571	*trpC2* Δ*tasA*::*cat*	Laboratory collection
GP1586	NCIB3610 Δ*tasA*::*cat*	GP1571 → NCIB3610
GP2034	*trpC2*Δ*pgpH::ermC*	Gundlach et al., 2015b
GP2040	*trpC2*Δ*gdpP::spc*Δ*pgpH::ermC*	Gundlach et al., 2015b
GP2047	NCIB3610 Δg*dpP::spc*Δ*pgpH::ermC*Δ*sinR::tet*	GP736 → GP2164
GP2160	NCIB3610 Δg*dpP::spc*	GP998 → NCIB3610
GP2161	NCIB3610 Δ*pgpH*::*ermC*	GP2034 → NCIB3610
GP2164	NCIB3610 Δg*dpP::spc*Δ*pgpH::ermC*	GP2034 → GP2160
NRS2450	NCIB3610 Δ*epsA-O::tet*	Ostrowski et al., 2011
TMB079	*trpC2* Δ*sinR::spc*	Jordan et al., 2007


### Assays of Complex Colony Formation

For the analysis of colony architecture, *B. subtilis* strains were pre-cultured in LB to an OD_600_ of 0.6–0.8. Five microliter of this cell suspension were then spotted onto minimal MSgg 1.5% agar plates (Diethmaier et al., 2011) and incubated at 30°C for 3 days. The colonies were photographed using an Olympus SZX12 stereomicroscope.

### Determination of SinR Protein Expression by Western Blot Analysis

To monitor the amounts of the SinR protein, the strains were grown in Spizizen minimal medium with glutamate and harvested in the logarithmic phase of growth (OD_600_ of 0.5). The cells were disrupted using a French press and 20 μg crude extract of each culture were loaded on a 15% sodium dodecyl sulfate-polyacrylamide gel. Following electrophoresis, the proteins were transferred onto polyvinylidene difluoride (PVDF) membranes (Bio-Rad) by electroblotting. Rabbit anti-SinR polyclonal antibodies served as primary antibodies. They were visualized by using anti-rabbit immunoglobulin alkaline phosphatase secondary antibodies (Promega) and the CDP-Star detection system (Roche Diagnostics), as described previously (Schmalisch et al., 2002).

### Transcriptome Analysis

Cells were grown in Spizizen minimal medium. Samples of wild type and *gdpP pgpH* mutant strains were harvested by centrifugation (10.397 × *g*, 1 min, 4°C) at mid exponential phase (OD_600_ of 0.5). A total of three independent biological replicates were included. The pellets were frozen immediately in liquid nitrogen and stored at -80°C.

RNA was isolated as described previously (Eymann et al., 2002; Nicolas et al., 2012). The quality of the RNA preparations was assessed by means of an Agilent 2100 Bioanalyzer according to the manufacturer’s instructions. Five microgram of total RNA were subjected to cDNA synthesis.

Synthesis and fluorescence labeling of cDNA followed a strand-specific method using the FairPlay III Microarray Labeling Kit (Agilent Technologies, Santa Clara, CA, USA) and actinomycin D (Calbiochem; Mäder and Nicolas, 2012). The individual samples were labeled with Cy5 and a reference pool containing equal amounts of RNA from each sample was labeled with Cy3. 100 ng of Cy5-labeled cDNA and 100 ng of Cy3-labeled cDNA were hybridized together to the microarray following Agilent’s hybridization, washing and scanning protocol (Two-Color Microarray-based Gene Expression Analysis, version 5.5). Data were extracted and processed using the Feature Extraction software (version 10.5). For each gene, the median of the individual probe ratios was calculated. Based on the common reference approach, these values represent relative gene expression levels of a given sample.

For statistical analysis, Genedata Analyst software (Genedata AG, Switzerland) was used. Genes with an FDR (false discovery rate) adjusted *P*-value less than 0.05 and at least 2.5-fold difference in expression levels between wild type and *gdpP pgpH* mutant were considered significantly affected.

### Real Time Quantitative Reverse Transcription PCR

For qRT-PCR, RNA isolation was performed as described above. cDNAs were synthesized using the One-Step RT-PCR kit (Bio-Rad) as described (Diethmaier et al., 2011). qRT-PCR was carried out on the iCycler instrument (Bio-Rad) following the manufacturer’s recommended protocol by using primer pairs for the analysis of *ptsH*, *epsA*, *tapA*, and *slrR* expression (for primer sequences see Supplementary Table S1 of Diethmaier et al., 2011). Data analysis and the calculation of expression ratios as fold changes were performed as described (Diethmaier et al., 2011). qRT-PCR experiments were performed in triplicate.

## Results

### Transcriptome Analysis of a Strain Accumulating c-di-AMP

To study the impact of cyclic di-AMP on the physiology of *B. subtilis* at a global level, we compared the transcriptomes of wild type strain 168 and strain GP2040 lacking both c-di-AMP specific phosphodiesterases (GdpP and PgpH). In a previous study, we have shown that this strain accumulates c-di-AMP, and that this accumulation is most significant when the cells were grown in minimal medium containing glutamate as the only source of nitrogen (2.5-fold accumulation; Gundlach et al., 2015b). Therefore, the bacteria were cultivated in Spizizen minimal medium with glutamate, and the RNA was extracted and analyzed.

The microarray analyses revealed that the levels of about 700 mRNAs were changed by the accumulation of c-di-AMP due to the deletion of the phosphodiesterase genes. Amongst the genes with most strongly increased expression levels, i e., more than 10-fold, were the *sunA* gene encoding the sublancin A precursor, and the *bmrCD* operon encoding a multidrug transporter. Most genes that respond with a strong reduction of their expression to c-di-AMP accumulation are mother-cell sporulation genes that depend on the sporulation-specific sigma factors SigE and SigK. Strikingly, the *tapA-sipW-tasA* and *epsA-O* biofilm operons as well as the *slrR* gene exhibit a strongly reduced expression upon c-di-AMP accumulation. A complete list of the mRNAs affected by the loss of c-di-AMP degrading phosphodiesterases is provided in the GEO database (accession number GSE78108) (see also **Supplementary Table S1**).

It has been observed previously that the expression of the SinR-controlled biofilm regulon and the SigD-dependent motility regulon are mutually exclusive (Cozy et al., 2012; Diethmaier et al., 2014). Indeed, many genes that are under the control of SigD are more strongly expressed in the strain accumulating c-di-AMP (about three- to sevenfold elevated expression). Expression of the *hag* gene encoding flagellin was increased fivefold in the absence of c-di-AMP degradation.

**FIGURE 1:**
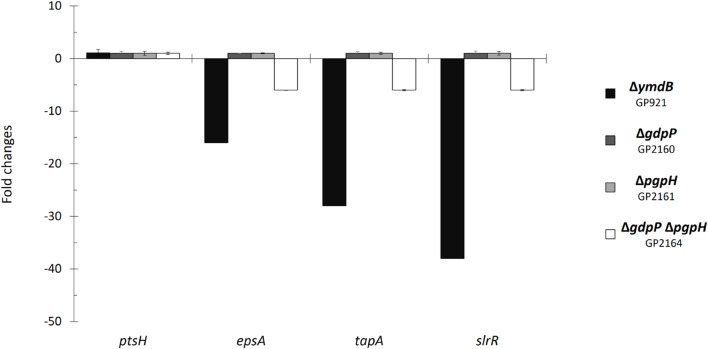
**Control of biofilm genes by c-di-AMP accumulation.** Fold changes in expression of *ptsH*, *epsA*, *tapA*, and *slrR* were investigated in the indicated mutant strains. The strains were grown in minimal medium supplemented with glutamate. RNA was purified from each strain, and quantitative RT-PCR was performed using primer sets specific to the indicated genes. Gene expression in the wild type strain NCIB3610 was set “1”. *ptsH* was used as a control. Errors bars represent the standard deviations of three replicates.

In order to verify the role of the c-di-AMP degrading phosphodiesterases in the control of biofilm genes, we determined the expression of representative genes by real-time quantitative reverse transcription PCR (qRT-PCR). Specifically, we tested *epsA* and *tapA* as the promoter-proximal genes of their operons as well as the *slrR* gene. Since the laboratory strain 168 is deficient for biofilm formation, we introduced all mutations into the background of the non-domesticated strain *B. subtilis* NCIB3610. To exclude any non-specificity of our assay system, we used the *ptsH* gene that is expressed under all conditions (Nicolas et al., 2012) as a control. Expression of this gene was not significantly affected by the mutations. As shown in **Figure 1**, the individual deletions of either phosphodiesterase gene did not affect the expression of the biofilm genes. In contrast the *gdpP pgpH* double mutant exhibited strongly reduced expression of all biofilm operons, thus confirming the results obtained in the transcriptome analysis.

### c-di-AMP Exerts Its Effect on Biofilm Formation via the Transcription Factor SinR

**FIGURE 2:**
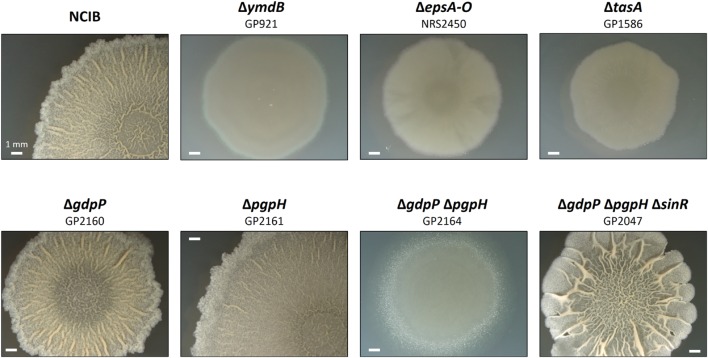
**Effect of phosphodiesterase deletion on biofilm formation.** Colony surface architectures of individual colonies grown on MSgg medium are shown. The colonies were filmed (stereomicroscope) after incubation for 3 days at 30°C. The wild type and mutant strains are as indicated.

The results presented above indicate that the accumulation of c-di-AMP may result in a defect in biofilm formation. To test this hypothesis, we tested the formation of complex colonies for a set of isogenic mutants that are all derived from the non-domesticated strain NCIB3610 (see **Figure 2**). In good agreement with previous observations (Kearns and Losick, 2005; Diethmaier et al., 2011), the wild type strain exhibited complex colony architecture. In contrast, strains carrying mutations known to affect biofilm formation (deletion of *ymdB*, the *epsA-O* operon or *tasA*) did not form complex colonies. Again, this result is in agreement with previous reports (Diethmaier et al., 2011; Gerwig et al., 2014). Deletion of the individual phosphodiesterase genes *gdpP* or *pgpH* did not affect colony architecture. The simultaneous deletion of both phosphodiesterase genes, however, resulted in loss of complex colony formation. Thus, the biofilm formation of the mutants faithfully reflects the expression of the biofilm genes: the single *gdpP* and *pgpH* mutants express the biofilm genes and form complex colonies, whereas both biofilm gene expression and complex colony formation are lost in the double mutant.

All biofilm genes that were found to be affected by the accumulation of c-di-AMP in this study are also members of the SinR regulon^1^ (see Chu et al., 2006; Michna et al., 2016). Therefore, it seemed possible that c-di-AMP exerts its regulatory effect on biofilm formation via the master regulator SinR. If this were the case, one would expect suppression of the defective biofilm formation by the inactivation of the *sinR* gene in the *gdpP pgpH* double mutant. Such a strain (GP2047) was constructed by phage transduction, and assayed for colony architecture. As shown in **Figure 2**, the deletion of *sinR* did indeed restore biofilm formation in a strain that accumulates c-di-AMP. These data demonstrate that c-di-AMP accumulation results in constitutive repression of biofilm genes by SinR.

**FIGURE 3:**
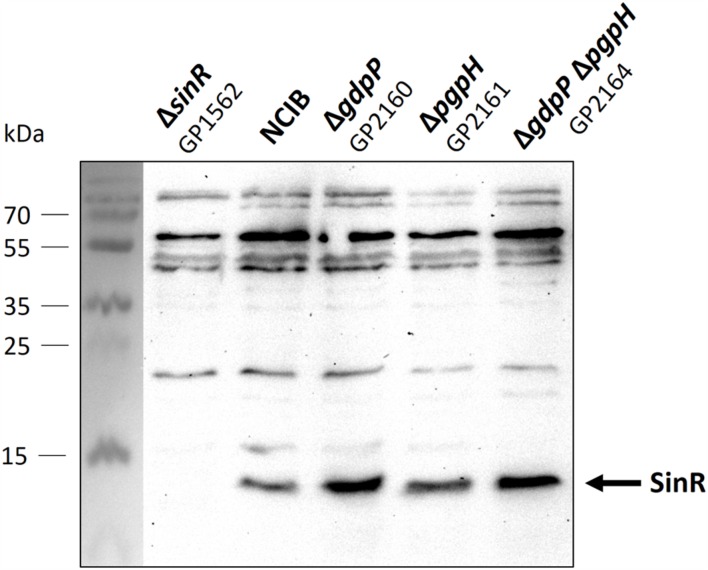
**Analysis of SinR protein levels in *Bacillus subtilis* NCIB3610 wild type and mutant strains.** Cells were grown in minimal medium supplemented with glutamate to mid exponential growth phase. Twenty microgram protein were separated using an SDS-polyacrylamide gel. Proteins were transferred to a PVDF membrane with standard Western blot technique and analyzed with antibodies raised against SinR. Strain GP1562 is a *sinR* mutant.

The increased repression of the SinR regulon upon c-di-AMP accumulation might result either from an increase in the cellular amounts of the SinR protein or from a larger fraction of the SinR protein pool that is available for transcription repression. To distinguish between these two possibilities, we determined the cellular amounts of the SinR protein in the wild type strain and the relevant mutant strains. For this purpose, the bacteria were cultivated to the mid-exponential phase in minimal medium supplemented with glutamate, and the SinR amounts were assayed by Western blots using antibodies raised against the *B. subtilis* SinR protein. As shown in **Figure 3**, the levels of SinR protein were not significantly affected by the deletions of the phosphodiesterase genes. Even the double mutant did not exhibit increased levels of SinR protein. These data suggest that c-di-AMP accumulation affects the activity of SinR rather than its cellular amounts (see Discussion).

## Discussion

c-di-AMP is an essential second messenger in *B. subtilis*, and the nucleotide was shown to be involved in a variety of biological processes such as cell wall metabolism and cell division, potassium uptake, central metabolism, and spore germination (see Commichau et al., 2015, for review). To extend our knowledge on the function(s) of c-di-AMP in *B. subtilis*, we have analyzed the global transcription pattern of a strain accumulating the nucleotide. Our results show that c-di-AMP accumulation affects the expression levels of a large number of genes, among them many sporulation genes and the genes required for biofilm formation. We have shown previously, that c-di-AMP formation by the sporulation-specific diadenylate cyclase CdaS is necessary for effective spore germination (Mehne et al., 2014). The observed effect on the expression of mother cell specific sporulation genes might reflect a specific role of c-di-AMP in sporulation-specific gene expression. This will be subject to further analysis.

Biofilm formation is a lifestyle of *B. subtilis* and many other bacteria that is subject to complex regulation. In *B. subtilis*, not only the master regulator SinR and its antagonists SinI and SlrR are involved in the control of biofilm gene expression, but also other factors. Among these are transcription factors like RemA and DegU, the phosphorelay that controls the phosphorylation state of the transcription regulator Spo0A, and the tyrosine protein kinases PtkA and EpsB (Kiley and Stanley-Wall, 2010; Vlamakis et al., 2013; Winkelman et al., 2013; Elsholz et al., 2014; Gerwig et al., 2014). More recently, control of the *sinR* mRNA stability by the endoribonuclease RNase Y was discovered as an additional important factor in the regulation of biofilm formation (Lehnik-Habrink et al., 2011; DeLoughery et al., 2016; Ogura, 2016). Finally, a phosphodiesterase, YmdB, is absolutely required for the expression of biofilm genes (Diethmaier et al., 2011).

With this study, we have extended the list of regulatory factors involved in controlling biofilm formation by the second messenger cyclic di-AMP. While c-di-GMP is known to be involved in the choice of lifestyles in many bacteria (Hengge, 2009; Römling et al., 2013), little is known about such a role of c-di-AMP. The investigation of functional roles of c-di-AMP is hampered by the fact that this molecule is both essential and toxic. Interestingly, there are very recent reports that implicate c-di-AMP in biofilm formation in the oral pathogen *Streptococcus mutans*. In this Gram-positive bacterium, c-di-AMP accumulation promotes biofilm formation (Peng et al., 2016). However, another study demonstrated that lack of c-di-AMP in *S. mutans* resulted in increased production of extracellular polysaccharides (Cheng et al., 2016). Clearly, further research is required to get the full picture of the role of c-di-AMP in *S. mutans* biofilm formation. Interestingly, the experiments with mutants lacking c-di-AMP-specific phosphodiesterases reported for *S. mutans* (Peng et al., 2016) and *B. subtilis* (this study) are contradictory. However, the specific regulation of biofilm formation in the two species differs strongly. While SinR is the master regulator in *B. subtilis*, this protein is not present in *S. mutans* or other streptococci. In contrast, c-di-AMP mediated signal transduction in biofilm formation in *S. mutans* involves the transcription factor VicR (Senadheera et al., 2005; Peng et al., 2016).

A major question to understand the role of c-di-AMP in the regulation of biofilm formation in *B. subtilis* is its relation to the master regulator SinR. In principle, c-di-AMP could act in the signaling chain upstream or downstream of SinR, or it could be part of a parallel regulatory pathway. Regulatory factors such as the response regulators DegU and Spo0A as well as the phosphodiesterase YmdB and the RNase Y act all upstream of SinR, i.e., they control the accumulation and/or the activity state of the SinR protein (Vlamakis et al., 2013; Diethmaier et al., 2014; DeLoughery et al., 2016). In contrast, the transcriptional activator RemA or the protein kinases PtkA and EpsB act independent of SinR (Kiley and Stanley-Wall, 2010; Winkelman et al., 2013). Two sets of experiments suggest that c-di-AMP accumulation in *B. subtilis* affects the activity of SinR: First, deletion of the *sinR* gene restores biofilm formation of the phosphodiesterase double mutant. Thus c-di-AMP acts in the same signaling chain as SinR, and the second messenger must be positioned upstream of SinR in this pathway. Second, c-di-AMP accumulation does not affect the cellular concentration of SinR, suggesting that the nucleotide might interfere with the interaction of SinR with its antagonists or affect SinR activity by a yet unknown mechanism. The control of transcription factors by second messengers is not unprecedented: a classical example is the control of carbon catabolite repression in enteric bacteria by the binding of cyclic AMP to its receptor protein Crp (Görke and Stülke, 2008), more recently activation of the *Streptomyces venezuelae* transcriptional regulator BldD was reported (Tschowri et al., 2014). Moreover, cyclic di-AMP was found to control the activity of the regulator protein DarR of *Mycobacterium smegmatis* (Zhang et al., 2013).

Very recently, glutamate and potassium homeostasis were implicated in the control of biofilm development in *B. subtilis* (Liu et al., 2015; Prindle et al., 2015). In *B. subtilis*, potassium uptake seems to be a major target of c-di-AMP-mediated signal transduction, and c-di-AMP accumulation is controlled by the nitrogen source (Commichau et al., 2015; Gundlach et al., 2015b). All these observations suggest the existence of an intricate network involving potassium homeostasis, nitrogen acquisition, and c-di-AMP signaling to regulate biofilm formation. Future work will have to identify the molecular mechanism(s) by which c-di-AMP controls SinR activity and thus biofilm formation in *B. subtilis*.

## Author Contributions

JG, UM, and JS designed the experiments. JG, HR, CH, and UM performed the experiments. JG, UM, and JS wrote the manuscript.

## Conflict of Interest Statement

The authors declare that the research was conducted in the absence of any commercial or financial relationships that could be construed as a potential conflict of interest.

## Footnotes

1 http://subtiwiki.uni-goettingen.de/wiki/index.php/SinR_regulon

## Supplementary Material

**TABLE S1 | Results of the transcriptome analysis.**
